# Enhancing the Cancer Care Journey for Indigenous Patients: A Guide for Oncology Nurses

**DOI:** 10.3390/curroncol32050279

**Published:** 2025-05-15

**Authors:** Jennifer M. Shea, Tina Buckle, Sylvia Doody, Kathy Michelin

**Affiliations:** 1 Division of Population Health and Applied Health Sciences, Faculty of Medicine, Memorial University, St. John’s, NL A1C 5S7, Canada; 2 Department of Health & Social Development, Nunatsiavut Government, Happy Valley-Goose Bay, NL A0P 1C0, Canada; tina.buckle@nunatsiavut.com (T.B.); sylvia.doody@nunatsiavut.com (S.D.); 3 Independent Researcher, Happy Valley-Goose Bay, NL A0P 1C0, Canada; kathymichelin@gov.nl.ca

**Keywords:** Inuit, indigenous health, cultural safety, health inequities, cancer journey

## Abstract

Background: Indigenous peoples nationally have seen a drastic increase in cancer diagnoses, often at later stages and with poorer survival rates than non-Indigenous Canadians. Colonization, assimilation policies, and racism within our healthcare system are contributors to these inequities. Methods: As a team, we have worked for over a decade to improve the cancer care journey of Indigenous patients in Labrador. We share learnings from a qualitative community-based project with Beneficiaries of the Labrador Inuit land claim agreement through sharing suggested improvements from participants to improve the cancer care journey. Objective: Acknowledging the diversity of Indigenous groups, we discuss suggestions as a guide and expand the discussion to provide interconnected suggestions for oncology nurses on enhancing care for their Indigenous patients. Conclusions: Oncology nurses play a crucial role in enhancing the cancer care journey for Indigenous peoples, necessitating a commitment to culturally safe environments, ongoing professional development, and advocacy for systemic changes.

## 1. Introduction

As a team of Indigenous (Doody and Michelin) and non-Indigenous (Shea and Buckle) healthcare professionals and researchers, we have worked together in Inuit cancer care for over a decade in Newfoundland and Labrador.

In our manuscript, we draw on our community-based participatory research with Nunatsiavut communities regarding the pre-diagnosis journey and share suggestions from participants on improving the cancer care journey. For this work, our roles were co-PIs (Doody, Buckle, and Shea) and project coordinator (Michelin). This manuscript was developed through a participatory approach, with the authors drawing on their lived experiences to contextualize and emphasize the originality of the methodology employed. In the following section we outline cancer experiences for Indigenous peoples in Canada to illuminate cancer care disparities.

## 2. Background and Context

The burden of cancer within Canada and globally has been well established. Cancer remains one of the two top causes of death for Canadians (shared with heart disease) [1]. Further, almost half of Canadians (45%) are projected to be diagnosed with cancer in their lifetime [2]. Strikingly, calculations of the potential years of life lost due to cancer mortality from 2018–2020 reached approximately 1.3 million years [1]. Nationally, the top cancer diagnoses include breast, lung, colorectal, and prostate [3].

Perhaps a lesser-known reality is how cancer affects certain groups within society, in particular, Indigenous groups. Indigenous peoples are defined in the Constitution Act, 1982, Section 35(2), as including the First Nations (Indian), Inuit, and Métis peoples of Canada [4]. In the 2021 Census, 1,807,250 individuals self-identified as having an Indigenous identity, accounting for approximately 5% of the Canadian population [4]. Cancer is a chronic disease that has become increasingly prevalent in First Nations, Inuit, and Métis populations within recent decades in Canada; it is now one of the leading causes of death for Indigenous peoples [5,6,7,8,9]. Many risk factors contribute to this increase in diagnosis for Indigenous Peoples, including a long history of colonialism, loss of culture, and dispossession of land [5,10,11].

Additionally, Indigenous peoples in the country present with later-stage cancers, which results in a high mortality rate from cancers that could have been prevented or effectively treated [12,13,14]. The impact of presenting with later stages of cancer has undeniable effects on the individual, family, and communities, as later stages come with additional challenges (e.g., prognosis). Many Indigenous peoples reside in northern, remote, and rural areas, impacting access to healthcare services and providers. Significant travel is required to access screening, follow-up, and treatment, which affects individuals’ families, employment, and support [15,16]. First Nations, Inuit, and Métis peoples identified three priorities in the Canadian Strategy for Cancer Control (2019–2029): (1) culturally appropriate care closer to home; (2) peoples-specific, self-determined cancer care; and (3) Indigenous-governed research and data systems [17]. While Indigenous communities face disparities, it is essential to approach the solutions from a strengths-based perspective. There is incredible resilience within communities, and from our personal experiences, we can attest that community-led approaches are crucial to moving forward in a positive direction.

### 2.1. Inuit and the Cancer Care Journey

Geographic isolation, food insecurity, housing conditions, and systemic inequities influence Inuit health outcomes. Of the three Indigenous groups in Canada, Inuit face the most significant burden when it comes to cancer morbidity and mortality [15,16]. For example, Inuit experience higher mortality rates for lung cancer compared to First Nations, Métis, and the general Canadian population [18]; furthermore, Inuit are now noted as having the highest rate of lung cancer globally [19]. Inuit populations in Alaska, the Northwest Territories, and Greenland are at an elevated risk of developing lung and colorectal cancer compared to the international average [19]. Rates of cervical cancer amongst Inuit are observed to be two to three times higher than the national average [20,21]. Further, there has been an increase in diagnoses of breast, lung, and colorectal cancers for female Inuit [20]. Historical impacts such as colonial policies, residential schools, forced relocation, and tuberculosis sanatoriums contributed to generational trauma and mistrust in Canadian systems, including healthcare [22,23]. Most communities in Inuit Nunangat are located in the north and are remote, resulting in geographical isolation that limits access to oncology specialists and treatment facilities. Furthermore, language barriers and the lack of culturally relevant resources within the healthcare system hinder effective communication and patient understanding. The underlying and interconnected impact of social determinants of health cannot be overstated, as they are direct contributors to health inequities [24,25].

### 2.2. Local Context

**Figure 1:**
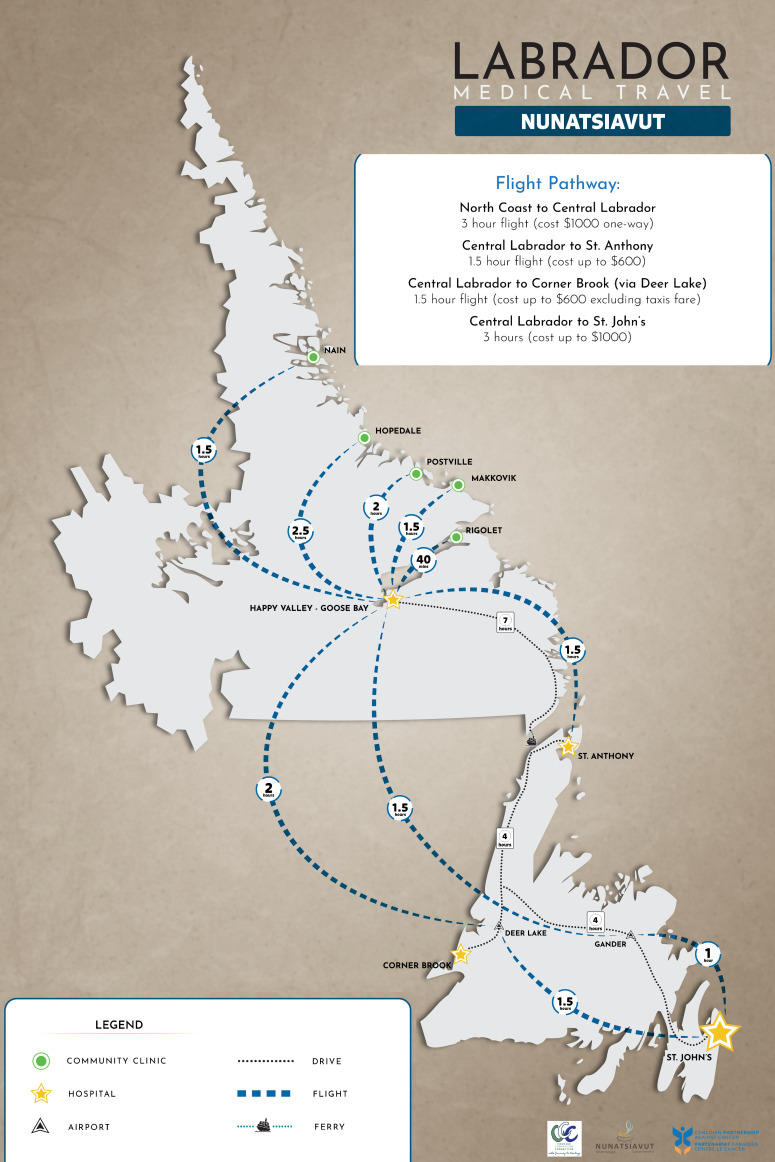
Nunatsiavut medical travel map: a visual aid was developed to educate healthcare professionals on the duration of travel for care. For a comprehensive map of Inuit Nunangat, please see [25].

Nunatsiavut is one of four regions in Inuit Nunangat. NG’s land claims area includes five communities (Nain, Hopedale, Postville, Makkovik, and Rigolet) on Labrador’s north coast. While each community has a clinic, services are limited at the community level, and travel is required for screening, diagnosis, and care. Similar to other jurisdictions (as tertiary care is often located in urban areas) there are no oncology care or specialists located in the five communities requiring travel for assessment, care, and treatment. Within the NG context, travel to St. John’s can take several days and is often interrupted by weather conditions. All five communities are fly-in only, with boat access available during the summer months. Figure 1 includes a travel map prepared as part of our project to highlight the distance and cost associated with travel.

## 3. Methods

A stakeholder session was held in Happy Valley-Goose Bay (HVGB) in 2018 to explore and discuss opportunities for improvement in the cancer journey and to develop a research proposal. Following this engagement session, the NG successfully obtained funding from the Canadian Partnership Against Cancer (CPAC) to partner with all Indigenous groups in Labrador and Memorial University (MUN) in exploring the pre-diagnosis cancer journey. The qualitative project “Courage, Compassion and Connection—The journey to healing: exploring cancer pre-diagnosis for Indigenous peoples in Labrador” explored the challenges and opportunities in the pre-diagnosis journey for Labrador’s Indigenous communities. For our project, we defined pre-diagnosis as the point at which the patient enters the healthcare system to receive preventative interventions or address symptoms, leading to further investigation.

In December 2019, a community planning session (pre-data collection) was held in HVGB to bring together communities to discuss the logistics of the data collection and provide advice. Each participating community had an identified community liaison who was the point of contact in the community for planning. During the community planning session, participants contributed to developing the final discussion guide to be used in the sessions (Table 1). Prior to data collection, research ethics approvals were obtained from the Health Research Ethics Board and NG.

The research design centered on storytelling methodologies, a traditional form of knowledge transfer for Inuit, allowing community members to share their experiences and expertise [26]. Sharing circles honour the voices of participants, position participants and researchers as equal, and adhere to the Indigenous methodology of story-telling [26,27]. While initially the research intended to employ only sharing circles, this was expanded to also include interviews following the COVID-19 pandemic to ensure participant comfort.

### 3.1. Participants and Recruitment

Through consultation with the NG, key participants were invited utilizing the identified community liaison. Participants included cancer patients/survivors, family members, community members, caregivers, and health professionals aged 16 and older. In design, we planned to have a sharing circle in each of the five NG communities, aiming for a total of 50 participants (10 per community). In total we had 32 participants (the number of participants in each community is marked in brackets after the community’s name). There were 26 female participants and 6 male participants. Data were collected from two sharing circles [Nain (8) and Hopedale (5)] and 18 interviews [Postville (9), Rigolet (8) and Makkovik (2)]. Participants included patients, family members, and healthcare providers. Due to the small number of participants and low populations in each community, no further breakdown of participants is provided to protect their privacy. Conversations were captured through audio recordings and note-taking, and audio files were transcribed verbatim. Participants were invited to review transcripts and any reports for accuracy. Each participant was provided a small token of thanks for taking the time to share their experiences consisting of a $25 gift card for a local store in the community.

### 3.2. Analysis

Transcripts were analyzed using Braun and Clarke’s [28] six-phase framework for thematic analysis: (1) become familiar with the data, (2) generate initial codes, (3) search for themes, (4) review themes, (5) define themes, and (6) write-up [28]. This framework was preferred due to the exploratory nature of the research questions. Shea and Michelin completed primary data analysis. Analysis progress was presented back to community leadership and liaisons face-to-face at two specific points, first during a retreat (August 2021) and second during the final project gathering (September 2022).

We identified six categories representing areas of improvement in the pre-diagnosis journey. These include: (1) prolonged investigation; (2) communication; (3) travel; (4) fear and anxiety; (5) be your own health advocate, and (6) access and supports. Table 2 lists the categories, their corresponding definitions, and an exemplary quote to provide further context. In the following section we focus on the suggestions offered by participants to improve the cancer care journey for Indigenous patients.

## 4. Improving the Cancer Care Journey for Indigenous Peoples

**Figure 2:**
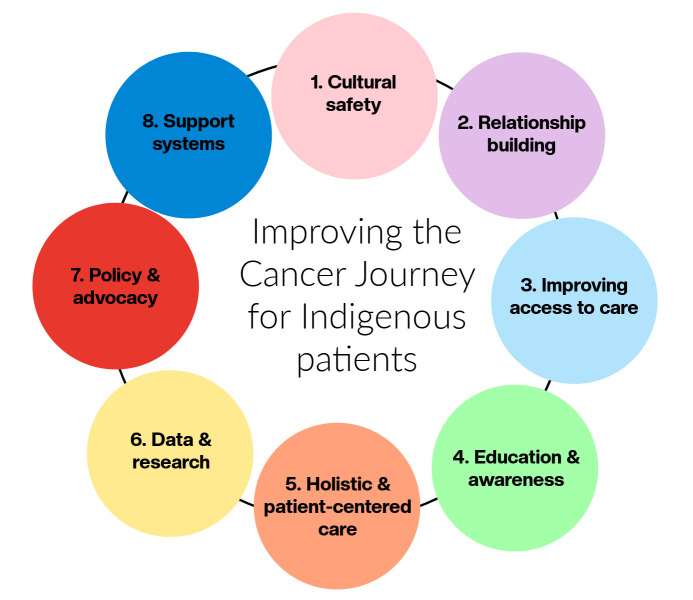
Enhancing cancer care for indigenous patients: a guide for oncology nurses, including eight actions that can be taken to improve the cancer care journey.

Nurses play a critical role in patient care and are often the most consistent healthcare providers patients encounter, both within and outside their community of residence. The barriers within our healthcare systems are vast and beyond the scope of one individual to change, but actions can be taken to improve care. First and foremost, we need to understand the history of Indigenous communities in Canada. While our collective knowledge of injustices has grown since TRC [29], and most recently in the discovery of unmarked graves for residential schools [30], for many Canadians, we did not learn about these truths in school, and it is critical to acknowledge that stereotypes and misunderstandings have continued to be perpetuated. In Figure 2, we identify suggestions that oncology nurses can take to improve the care journey for Indigenous patients. While these will subsequently be described as individual actions, they are all connected and may overlap.

### 4.1. Cultural Competence and Safety

Participants in our study shared instances of discrimination as Indigenous peoples and spoke about the impact of stereotyping (e.g., result of lifestyle factors such as drinking and smoking) they experienced while receiving care. Cultural safety was developed over 30 years ago in New Zealand through nursing practice as a framework to address health inequities experienced by the Māori people [31]. It emerged in response to the recognition that traditional healthcare systems were failing Māori due to systemic racism, cultural insensitivity, and a lack of understanding of Māori worldviews and health practices [32]. To expand knowledge, consider training opportunities to understand the historical, social, and cultural contexts of Indigenous peoples, including the impact of colonization, residential schools, and systemic racism. Through a commitment to cultural safety, the focus becomes on creating an environment where Indigenous patients feel respected, safe, and understood. What differentiates cultural safety from competency is that it involves self-reflection on biases and power imbalances in healthcare settings [32]. Without an understanding of the impact of racism in healthcare, providers are unable to work on addressing root causes. An understanding of cultural safety provides a critical foundation for addressing inequities.

### 4.2. Building Trust and Relationships

The challenges faced by Indigenous patients regarding cultural safety underscore the importance of building relationships between them and healthcare professionals. Participants in our study discussed the importance of relationship building and noted discomfort when they do not feel safe within the healthcare system. Nurses can establish trust through consistent, respectful, and empathetic communication. Recognize that mistrust of the healthcare system may exist due to historical and ongoing injustices and that building trust and relationships may take time. Nurses can consider collaborating with Indigenous communities and leaders to develop and implement culturally relevant, community-driven cancer care programs. Establishing trust and relationships is critical to achieving cultural safety for Indigenous patients [31]. In a Saskatchewan study, Indigenous participants emphasized the importance of strengthening relationships between the community and the health system to ensure responses align with their needs and foster culturally appropriate care and services [33].

### 4.3. Access to Care

As our study focused on a northern and remote region, access to care was a significant barrier for participants. Given the vast geography of our country and the concentration of advanced healthcare settings in urban areas, it is essential to be mindful and aware of access barriers patients can face. A national survey with oncology nurses explored understanding of access to cancer care for Indigenous patients and found that while nurses perceived themselves as mediators of access to care, they are often constrained in what they do through the biomedical model of practice [34]. Often, nurses would speak of individual barriers (e.g., transportation), while structural challenges (e.g., racism within the healthcare system) were frequently overlooked, reflecting the biomedical model of health [34]. Awareness of Indigenous history, as well as definitions of health and the social determinants of health, is critical for transitioning to person-centered care. Moving forward, it is essential to address barriers such as geographic isolation, transportation issues, and financial constraints in collaboration with Indigenous governments and communities. Nurses can advocate for policies that improve access to cancer screening, diagnosis, and treatment, as well as telehealth services, mobile screening units, and culturally adapted oncology resources. While cancer patient navigators are widely used throughout the country, Indigenous Patient Navigators are not as well-established in our systems. Indigenous Patient Navigators play a critical role in ensuring cultural safety and can provide language interpretation to enhance patient understanding. A recent review of cancer patient navigators has highlighted the importance of this service, not only for the individual but also for strengthening the healthcare team’s performance and fostering trust with patients [35].

### 4.4. Education and Awareness

In our study with Inuit participants, the need for enhanced education on cancer types and symptoms was noted as a valuable addition. It was suggested that these education sessions could be hybrid, including utilizing healthcare professionals hired by the Indigenous government, survivor stories, visiting speakers, and virtual options. Partnership with Indigenous governments and communities would be essential to ensure the need and coordination of translation services if needed. When developing materials, it is vital to provide clear and accessible information about cancer prevention, screening, and treatment options. In a study with Indigenous cancer survivors in Saskatchewan, Indigenous Patient Navigators were identified as key in supporting patients with medical interpretation, translation, and education [36]. Oncology nurses can partner with Indigenous Patient Navigators to deliver and develop culturally safe education sessions and materials that benefit Indigenous patients throughout their journey. National working or special interest groups would be beneficial to share experiences and lessons learned across jurisdictions. Locally, nurses can participate in outreach programs to raise awareness about cancer risks and the importance of early detection, working in partnership with Indigenous communities. Indigenous peoples have noted the need for enhanced understanding and education regarding cancer to address fear and anxiety [17].

### 4.5. Holistic and Patient-Centered Care

Participants noted that the western healthcare system does not match Indigenous ways of knowing and wellness, in turn complicating the healing journey. Healthcare professionals receive minimal education on healing beyond the biomedical scope, thus requiring enhanced integration of knowledge keepers, traditional healers, and elders into the system [37]. Nurses should tailor care plans to the individual needs and preferences of Indigenous patients, considering their cultural, spiritual, and emotional well-being. Modification of care plans can be attained through connections with Indigenous Patient Navigators, elders, community health representatives, and local support groups. We must recognize the importance of Indigenous healing methods and integrate and advocate for them where possible for the patient’s benefit. A study with First Nations communities highlighted the need for holistic healthcare that respects traditional healing practices and acknowledges the historical trauma experienced by these communities [38]. This can be achieved through the de-stigmatization of traditional healing, fostering trust, and ensuring access to traditional healing and medicine within the healthcare system. Oncology nurses in particular can advocate for integration of traditional approaches and connecting with experts (e.g., elders) who can help facilitate.

### 4.6. Data and Research

For NG, it is critical that research they participate in is of benefit to communities and completed in partnership. Our collective work was designed and carried out in partnership from beginning to end. In our development phase, we also prepared a Memorandum of Understanding (or research agreement) outlining the roles and responsibilities of each partner. Indigenous communities are diverse, so the identification of issues and the development of responses need to be tailored accordingly. Within local settings, nurses can support efforts to collect and analyze data on cancer incidence, outcomes, and care experiences among Indigenous populations to identify gaps and inform policy. A recent scoping review examining cancer care for rural and remote populations highlighted the lack of patient and community engagement in research [39]. To encourage and facilitate Indigenous participation in cancer research, it is important to ensure that studies are relevant and beneficial to communities. It is crucial that any research undertaken is conducted in partnership with the Indigenous community and adheres to protocols such as OCAP© [40], the National Inuit Strategy on Research [41], and the Principles of Ethical Métis Research [42]. This ensures that the work proceeds in a manner that is respectful and beneficial to Indigenous patients and communities.

### 4.7. Policy and Advocacy

Participants shared with us that policy often limits the ability to engage in traditional healing (e.g., smudging or lighting of the kullik in healthcare facilities). Previous studies have found that oncology nurses are constrained from providing culturally safe and trauma-informed care by the organizations and systems in which they work [43]. In our previous experiences, we have found that creating teams, including healthcare leadership, has fostered change and contributed to the sustainability of developed responses. Nurses can advocate for systemic changes that address health inequities and improve cancer care for Indigenous populations, including funding for Indigenous-led health initiatives and equitable resource allocation. As a first step, policies, procedures, and organizational standards should be reviewed to identify areas where changes can be made [43]. Through research projects, evidence can be gathered to highlight where and how changes are required to achieve cultural safety for Indigenous patients. Nurses must engage in continuous professional development to stay informed about best practices in Indigenous health and oncology care. Patient advisory groups are often utilized in healthcare settings; the creation of a group focusing on Indigenous patients can help direct and ensure movement in the right direction.

### 4.8. Support Systems

Participants in our project spoke at length about the lack of on the ground supports and across multiple communities, and the idea of support groups was suggested as a tangible and cost-effective solution. Supports provided to patients likely vary based on jurisdictions, but standard support from a dietician or social worker is typically standard practice. A recent systematic review found that virtual health interventions to reduce travel barriers can improve patients’ access to screening, care, treatment, and support [44]. Nurses can consider providing access to counselling, support groups, and other resources that address the emotional and social impacts of cancer. Given the geographic dispersion of communities, virtual options or localized supports should also be considered. As there may be limitations to technology and access, virtual care options must be developed, implemented, and evaluated in partnership with Indigenous groups [45]. Virtual care became more common following the COVID-19 pandemic, with research suggesting that patient satisfaction increases when options extend beyond telephone use to connect with patients [46].

## 5. Conclusions

We have shared recommendations suggested by participants in our study with Nunatsiavut Inuit in Labrador. The reflections from this region may not be representative of all Indigenous groups in Canada. Rather, we hope that outlining suggestions can be beneficial to other areas and healthcare professionals as they seek to enhance care for Indigenous patients. By incorporating Indigenous perspectives into oncology care, nurses can contribute to better cancer outcomes and community well-being. Improving cancer care for Indigenous patients in Canada requires a multifaceted approach that addresses systemic barriers, cultural sensitivities, and health disparities. Nurses in oncology must be equipped with specific knowledge and skills to provide effective and culturally safe care. One of the most important steps that nurses can take is connecting with local Indigenous communities and governments in their area to learn about gaps, strengths, and develop relationships. In urban settings, Friendship Centres can be an invaluable resource and often work with healthcare systems (e.g., training, accommodations, and navigators). Our manuscript outlines eight considerations arising from the participants, including cultural safety, relationship building, improving access to care, education and awareness, holistic and patient-centered care, data and research, policy and advocacy, and support systems. By focusing on these areas, oncology nurses can contribute to reducing health disparities and improving cancer outcomes for Indigenous patients in Canada. Collaboration with Indigenous communities, continuous learning, and a commitment to cultural safety are essential to this effort.

## Figures and Tables

**Table 1:** Discussion guide for sharing circle and interview discussions.

Patient	Family/Caregivers	Healthcare Professionals
Have you ever participated in any screening initiatives before?	Do you (or have you) help care for someone with cancer?	What is your role in a patient’s cancer journey?
Are you aware of any screening tests available in your community?	Are you aware of the patient’s journey to diagnosis?	Are there any experiences you would like to share regarding in caring for a cancer patient?
How did you enter the healthcare system?	How were you involved with the patient leading up to a diagnosis?	What are two (2) things that you think works within the system?
Were you happy with the services and communication that were provided?	What are two (2) things worked well and that you were happy with?	What are two (2) things you would change to make the process better?
What are two (2) things worked well and that you were happy with?	What two (2) things would you have changed to help make the process better?	
What two (2) things would you have changed to help make the process better?	Please tell me about the journey. Include what you felt as well.	
What supports were available to you? (e.g., stress, uncertainty etc.…)	Is there anything else that you would like me to know?	
Is there anything else about your pre-diagnosis cancer journey that you would like me to know?		

**Table 2:** Categories for improvement, definitions, and exemplary quote—courage, compassion, and connection.

Improvement Area	Definition	Exemplary Quote
Prolonged investigation	Captures the drawn-out and often fragmented diagnostic journey experienced by Indigenous individuals prior to a formal cancer diagnosis. It encompasses delays, missed diagnoses, and systemic oversight, where patients often felt they were not being taken seriously or were navigating a system unequipped to recognize or address their concerns in a timely manner.	“So he takes this on himself this time to go to the clinic—“I threw up a couple of times and I don’t feel right”. Anyway, the nurse here was a really dedicated nurse…it went to the nurse first time sending him out because she felt there’s something on the go here…they send him back with a chest infection, before the pills run its course, then he encounters some throwing up, then the nurse sends him back out, they send him back with pneumonia…upon the second time he arrived home, six weeks later, we were standing in the church at his funeral. No more than a week after his funeral, the results of his second trip—the real results, I guess, all of them—came back. And one of the results was he had a huge tumor in his stomach. How can this go unnoticed?"
Communication	Highlights the role of interpersonal and systemic communication, including both barriers and facilitators. It includes experiences of language differences, medical jargon, stereotyping, and cultural misunderstandings, alongside moments where healthcare providers either enhanced or hindered understanding and trust	“With technology these days, that should not be an issue. They should have access even if it’s online to an interpreter to translate for the doctor and the patient. Just to ensure that the patient does understand because a lot of times the terminology and certain way things are said in context—in Inuktituk and English—are translated totally differently…And with the severity… when it’s imminent that you get the right translation, especially with cancer being that it’s a terminal disease, can be a terminal disease. And to ensure that the patient clearly understands what they’re going to face and what they’re facing. I don’t think there should be a question whether that patient understood.”
Travel	Relates to the physical and emotional toll of the travel required to access care. Many Indigenous patients must leave their communities, incurring costs, enduring disruption of family life, and facing logistical challenges that impact timely diagnosis and continuity of care.	“And the resources really need to be visited though because when you go away and there’s everything right there, and I know we’re isolated but we don’t have enough services. You got to go so far away even just to get an appointment for X-Ray. You can go days and days without your family and your work. People can go and get an X-Ray and be off for half an hour. We go away and we’re gone for three days, maybe longer. How come there are no resources on the ground and in the communities, and we are having to go away. Very important. Health care services alone.”
Fear and anxiety	Focuses on the emotional burden that precedes diagnosis, such as anticipatory grief, family history of cancer, and trauma linked to past experiences with healthcare.	“A lot floods back because we’ve survived a lot of cancer. And there’s so many more stories and it hasn’t changed a whole lot since I had cancer. And to see people who have been diagnosed terminally, you wonder what they are doing for them when even as survivors, we had to struggle with that and try to deal with it the best way you can. And it really does change who you are. For those that are fighting to the end, I can’t imagine how hard it must be for them. Even just to think of, “Okay I got cancer. What if it gets really bad? I might die from this.”
Be your own health advocate	Represents the individual resilience and proactive behaviours needed to navigate an often unresponsive or dismissive healthcare system. It includes recognizing one’s own symptoms, demanding attention, and persisting despite barriers.	“I find that…until you push, and say I’m not leaving here until something is done, then they dismiss you and say well come back in 6 months, if you’re still having the same symptoms, you know we’ll deal with it, we’ll do some more scans, but they don’t really get to the root of it unless you say I’m not leaving without something being done. And most of our people, our people like we don’t do that.”
Access and supports	Focuses on the structural availability of healthcare services, including gaps in local care, limited Indigenous-specific services, and navigation challenges. It also includes both the presence and absence of supportive roles, such as patient navigators and community advocates.	“The journey is hard because it’s like trying to navigate and trying to find supports and not knowing who to ask what to do, or what can I do to get better service, you look through all those things because when you try to refer yourself to someone, it comes back. They used to come back to me and say, “You can’t refer yourself. You’ve got to be referred out by a nurse or a doctor”. You can’t win, you couldn’t win at that time, even with something as serious as cancer going on. And my dad, he was in his 50s. They had diagnosed him with pneumonia and sent him out. He never came back. He had lung cancer. But he died after"

## Data Availability

As an Indigenous health research project, data are the property of the Nunatsiavut Government and are not publicly available.
